# A retrospective analysis of peri-operative medication errors from a low-middle income country

**DOI:** 10.1038/s41598-022-16479-7

**Published:** 2022-07-20

**Authors:** Shemila Abbasi, Saima Rashid, Fauzia Anis Khan

**Affiliations:** Department of Anaesthesiology, Aga Khan University Hospital, Karachi, 74800 Sindh Pakistan

**Keywords:** Health care, Medical research

## Abstract

Identifying medication errors is one method of improving patient safety. Peri operative anesthetic management of patient includes polypharmacy and the steps followed prior to drug administration. Our objective was to identify, extract and analyze the medication errors (MEs) reported in our critical incident reporting system (CIRS) database over the last 15 years (2004–2018) and to review measures taken for improvement based on the reported errors. CIRS reported from 2004 to 2018 were identified, extracted, and analyzed using descriptive statistics and presented as frequencies and percentages. MEs were identified and entered on a data extraction form which included reporting year, patients age, surgical specialty, American Society of Anesthesiologist (ASA) status, time of incident, phase and type of anesthesia and drug handling, type of error, class of medicine, level of harm, severity of adverse drug event (ADE) and steps taken for improvement. Total MEs reported were 311, medication errors were reported, 163 (52%) errors occurred in ASA II and 90 (29%) ASA III patient, and 133 (43%) during induction. During administration phase 60% MEs occurred and 65% were due to human error. ADEs were found in 86 (28%) reports, 58 of which were significant, 23 serious and five life-threatening errors. The majority of errors involved neuromuscular blockers (32%) and opioids (13%). Sharing of CI and a lesson to be learnt e-mail, colour coded labels, change in medication trolley lay out, decrease in floor stock and high alert labels were the low-cost steps taken to reduce incidents. Medication errors were more frequent during administration. ADEs were occurred in 28% MEs.

## Introduction

Medication error reporting is considered an essential part of improvement strategies for patient safety and quality of care. The World Health Organization (WHO) in their third global safety challenge aimed to reduce the global burden of iatrogenic medication-related harm by 50% within 5 years^1^. The three priority areas of medication safety mentioned by them were high-risk situations, polypharmacy, and transition of care. Peri operative anesthetic management of patient encompasses all these three areas. Anesthesiologists are responsible for multiple drug prescription, preparation, dilution, administration, and documentation and monitoring of medication during the perioperative period.

Several publications have reported these errors from high income countries (HIC)^2^ and high middle-income countries but there is limited published data from low-and middle-income countries (LMICs)^2,3^. There are multiple factors responsible for scarce data from LMIC, a great challenge for healthcare providers is recognizing and reporting medication errors. In addition, many factors such as health providers’ workload, lack of reporting system, education, training, institutional policies and protocols, and fear of disciplinary actions are responsible for under reporting^4^ There is a culture of blaming, lack of knowledge regarding impact of reporting, and lack of training on how to report errors are the issues that lead to under-reporting of medication errors by health care providers^5^.

Furthermore, there is relatively scarce data on medication errors in the lower-middle-income countries (LMIC) as Egypt, Palestine, Syria, Yemen, and Iraq^6^. In last two years literature regarding increasing awareness of medication error and patient safety are published from LMIC^7,8^. Medication errors (MEs) are voluntarily reported as part of our departmental Critical Incidents Reporting System (CIRS). Khan et al. previously reported on critical incidents from our department, occurring between 1997 to 2002, and found that one fifth of reported incidents were related to medication^9^. In last two decades a lot has happened within the department in terms of processes of medication and equipment.

Our study had two objectives: (1) to identify, extract and analyze the medication errors (MEs) reported in our critical incident reporting system (CIRS) database from 2004 to 2018; (2) to review the actions taken for improvement based on the reported errors.

## Methods

The Ethical Review Committee (ERC) of the Aga Khan University waived the requirement for informed consent for this study (ERC no. 2020-3421-8389). In addition, the protocol was reviewed and approved by the Departmental Research Committee. All methods were performed in accordance with the relevant guidelines and regulations. It is a retrospective cross-sectional study. This monocentric study was conducted at department of Anaesthesiology at a tertiary care hospital.

All critical incidents (CI) reported from 1st January 2004 until December 2018 related to medication errors in adult patients aged 18 and above, were retrieved and then reviewed independently by two authors (SABB and SIRH). In the initial review authors divided the medication errors as follows; errors of medication selection/planning or ordering, dispensing, preparing, administering, documenting, and monitoring, using the operational definitions published by Nanji et al.^3^. All relevant information was entered in specially designed data extraction forms. The extracted data also included year of reporting, patients age, surgical specialty, ASA status, time of incident, phase and type of anesthesia, phase of drug handling, type of error, class of medication, outcome in terms of level of harm, severity of adverse drug event (ADE) distinction and any steps taken or that needed to be taken for improvement.

The distinction of type of errors into human error, system error, and equipment error was already present in the Critical Incident Review (CIR) forms available in the system.

The outcome of these errors was then graded by the reviewers into errors with no harm, little potential for harm, potential for ADE, and ADEs. ADE were further divided into significant errors (minor physiological disturbance), serious (major physiological disturbance) and life-threatening (morbidity or mortality)^3^. If an ADE had occurred due to a known allergic reaction it was classified as “no error “.

A second review was conducted to observe any disparity between the two reviewers. A third investigator was consulted (FK) if the discrepancy was not resolved between the two initial reviewers. Data was then entered in Statistical Package of Social Sciences version 19.0 (S.P.S.S.). Descriptive analysis was carried out to report the error categories and types. Fisher exact test was applied to explore any association of error category, type of errors, and outcome with demographic variables.

## Results

During the study period (2004–2018), 1006 critical incidents were reported in 201,111 procedures, in adult patients (age > 18 years) undergone anesthesia and surgery. Our initial review identified 336 medication errors. As 25 forms were excluded for not fulfilling the criteria based on the operational definitions used. Disagreements were found in 15 forms which were reviewed and resolved by third investigator, providing 311 medication errors for analysis.

As far as demographic and clinical variables are concerned, the highest number of errors were reported as: in age group 39–48 there were 82 (26.4%) MEs, 66 (21%) in Ear Nose Throat (ENT) specialty, 163 (52.4%) in ASA status II, 133 (42.8%) at induction of anesthesia, 267 (85.9%) in general anesthesia, and 268 (86.2%) during office hours i.e., 8 a.m–5 p.m. The frequency (%) of medication errors corresponding to the demographic and clinical variables are shown in Table 1.

**Table 1:** Demographic and clinical variables.

Demographic variables	f (%)	Clinical variables	f (%)
**Age groups (years)**		**Surgery specialty**	
19–28	45 (14.5%)	ENT	66 (21.2%)
29–38	54 (17.4%)	Neurosurgery	62 (19.9%)
39–48	82 (26.4%)	General surgery	44 (14.1%)
49–58	45 (14.5%)	Orthopedic	32 (10.3%)
59–68	44 (14.1%)	Urology	29 (9.3%)
More than 68	41 (13.2%)	Gynecology	29 (9.3%)
**ASA**		**Phase of anesthesia**	
I	39 (12.5%)	Induction	133 (42.8%)
II	163 (52.4%)	Maintenance	122 (39.2%)
III	90 (28.9%)	Emergence	15 (4.8%)
IV	19 (6.1%)		
**Timings**		**Type of anesthesia**	
Office hours (8a.m-5p.m)	268 (86.2%)	General anesthesia	267 (85.9%)
Other	43 (13.8%)	General and regional	12 (3.9%)
		Spinal	21 (6.8%)

Medication errors involved 13 different drugs categories administered in the perioperative period. Major drugs categories were 102 (32.8%) neuro-muscular blockers, 41 (13.2%) opioids, 34 (11%) sedative/hypnotics, 32 (10.3%) vasopressors and 18 (5.8%) local anesthetics. The most common medications reported in these categories were Atracurium, Fentanyl and Pethidine, Midazolam, Phenylephrine, and Bupivacaine respectively.

On analyzing the categories of medication errors, 188 (60.5%) reported during administration, 67 (21.5%) during preparation and 39 (12.5%) during dispensing were the main contributing categories. Selection, documentation, ordering, and monitoring were 8 (2.6%), 6 (1.9%), 3 (1%) and 0 incidents respectively. Commonly occurring incidents from major contributing categories with their frequency (%) and action taken to bring improvement in system are shown in Table 2. Percentages are calculated from respective total. It was observed that the most common administration error was “Overdose” in 38 (20%) reports followed by “Ineffective neuromuscular blockers” in 36 (19%) and “Wrong medicine administered” 34 (18%). For instance, overdose of Injection Phenylephrine happened in many cases in a row, root cause analyzed and immediate action taken as shown in Table 2. Wrong medicine was due to syringe swap; Atracurium was given instead of Midazolam.

**Table 2:** Medication errors during drug administration, preparation, and dispensing along with corrective measures taken (2004–18).

Medication errors	f (%)	Corrective steps taken
**Administration errors**	**188**	
Overdose	38 (20%)	Emphasis on dose calculation while making anaesthesia plan Prefilled syringes of vasopressor (phenylephrine and epinephrine) were discontinued from pharmacy
Wrong medicine administered	34 (18%)	Re-enforcement of SSP
Ineffective NMBs	36 (19%)	Pharmacy informed to ensure cold chain maintenance. Vendor changed and new NMBs added in formulary
Under dosage	23 (12%)	Emphasis on dose calculation while making anaesthesia plan
Side effects	20 (11%)	Discussion in departmental meeting and dissemination through “Lessons to Learn” email. A separate training session for new trainees proposed to residency committee
**Preparation errors**	**67**	
Labelling errors	40 (60%)	In year 2007, printed color-coded labels were introduced for cardiac medications, in 2010 for induction agent, muscle relaxant, opioids, and local anaesthetic and in 2018 for all medication
Dilution errors	11 (16%)	This information was shared in CI meeting and followed by lesson to learn email. Reminder on drug stations, “BREAK and MAKE one by one.”
Deviation from SSP	11 (16%)	Re-enforcement of SSP in CI meetings and during on job training
**Dispensing errors**	**39**	
Ampoule swaps	11 (28%)	Floor stock (quantity and variety) was decreased in 2007. Medication trolley and floor stock checking in every shift (thrice a day) in 2007
Wrong medication	8 (21%)	Cross checking of labels and ampoules before receiving from pharmacy LASA (Look Alike and Sound Alike) medicine identification and labeling from year 2018 by the pharmacy
Syringe swaps	5 (13%)	Re enforcement of “READ OUT LOUD” before injecting any medicine or connecting any infusion. Standardized lay out in medication tray, workspace, and drug trolley
Non-compliance to narcotic handling	4 (10%)	Narcotic policy, POE of narcotics and its dilution by pharmacy started in 2014
Expired drugs on drug trolley	3 (8%)	In addition to other checks regular audits of whole drug trolley by pharmacy representative at all locations, for correct medication in correct location. Check of its expiry and any breakages. Near expiry (6 months) medicines withdrawn if present

*SSP* Syringe Standardization Policy, *NMB* Neuromuscular blocker, *POE* Patient Order Entry.

The errors were also classified as 204 (35.6%) human errors, 68 (22%) system errors and 9 (2.9%) equipment errors. In 30 reports there was no error, but two common findings were observed; in 12 reports, known allergic reaction to the anesthetic medication without any history of allergy in those patients while in 11 cases, inefficacy of medicines (Atracurium, Bupivacaine, and Succinylcholine) were the reason to report. On further analysis of 204 human errors, lack of either knowledge, judgement, or check, was found in 105 (51%) reports, deviation from standard practice in 65 (32%), stress factor in 18 (8.8%), and poor communication in 16 (7.8%) reports. Furthermore, lack of check was observed in 50 (47.6%), lack of judgment in 36 (34.2%), and lack of knowledge in 18 (17%) reports.

**Figure 1:**
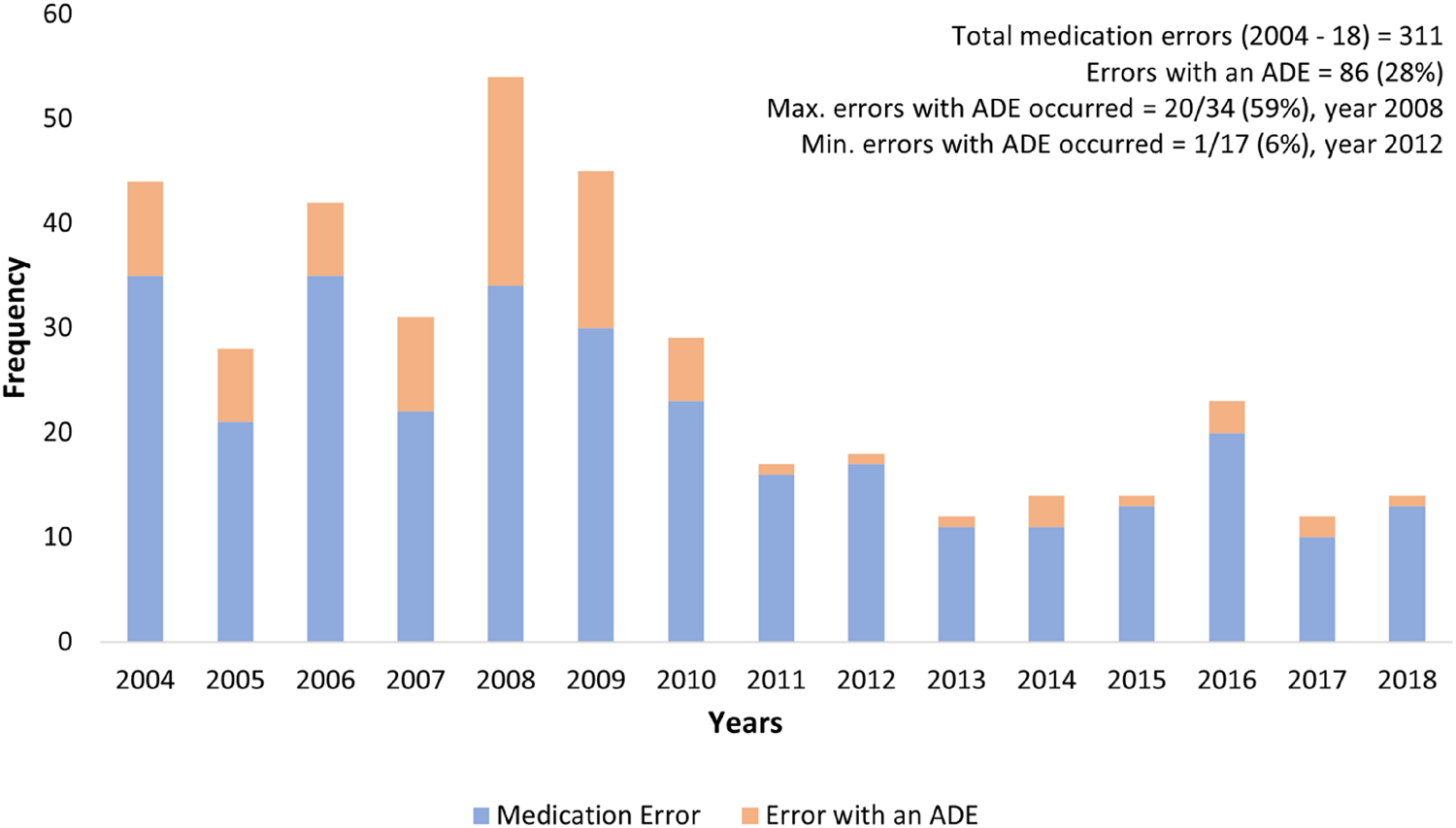
Comparison of medication errors with Adverse Drug Event (2004–18).

The medication errors were also assessed for any harm or ADE. The outcome was as 88 (28.3%) errors with no potential for harm, 61 (19.6%) errors with little potential for harm, 33 (10.6%) errors with little potential for ADE, 86 (27.7%) errors with an ADE, 30 (9.6%) ADE without error, and 13 (4.2%) errors with potential for ADE. Out of 86 errors with an ADE, the severity was observed as 58 (67.4%) significant, 23 (26.7%) serious and 5 (5.8%) life threatening in reported MEs. The year wise comparison of medication errors with corresponding number of errors with ADE are displayed in Fig. 1, which gives a picture of decrease in number of ADEs in comparison to previous years.

**Figure 2:**
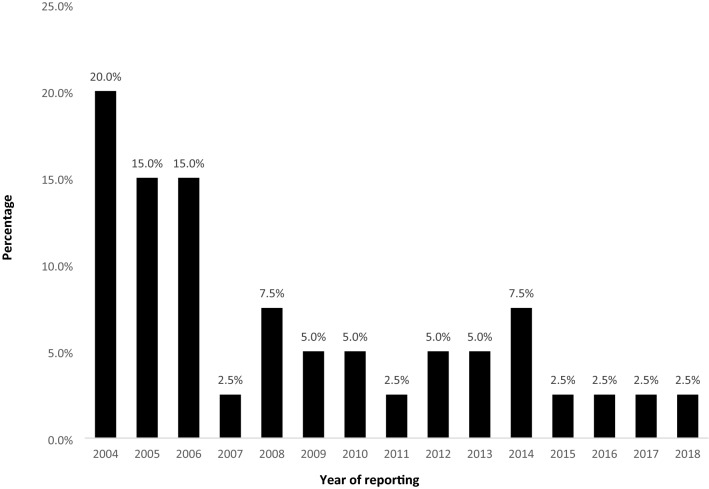
Percentage of labelling errors during medication preparation (2004–2018).

Some of the strategies that were recommended and were put in place to manage 23 serious errors with an ADE are shown in Table 3. Effects of one of these strategies i.e., transition from handwritten labels to color-coded labels started in 2007 as shown in Table 2 and Fig. 2. Finally, three demographic variables (age groups, ASA, and timing) were analyzed for any association with errors category, type of errors, and outcome by using Fisher Exact test. There was statistically significant association between age groups and category of error (p-value 0.014), age groups and type of error (p-value 0.009), and timings and type of error (p-value 0.002).

**Table 3:** Causes and corrective measures taken for serious medication errors with ADE (2004–18).

Incident	n = 23	Cause/immediate action	Corrective measure/awareness created
Patient found hypotensive when received in the operating room	1	Patient shifted to OR after receiving Inj. Hydralazine 15 mg I.V. without any monitoring. Monitoring initiated and Hypotension was treated	Transfer policy was redesigned that sick patients should not be shifted to or from OR without standard monitoring
Epinephrine infusion started because of severe hypotension (70/50, 50/35, 45/35 mmHg)	1	Faulty BP apparatus which showed severe hypotension, no effect of vasopressors seen. On change of BP apparatus BP was 180/110 mmHg. Epinephrine infusion stopped	Regular calibration of BP apparatus at the beginning of list Always think about faulty equipment in case of erratic reading
Bupivacaine infusion of 0.125% dispensed instead of 0.0625% entered in POE Nurse started it at the rate of 15 mls/h	1	POE not followed by nurse and pharmacy. Correct infusion started when error discovered	Sharing of incident at CI meeting Stressed upon following POE system
Severe hypertension (210/110 mmHg)	1	Fentanyl given in incremental doses up to 300ug, but no response noticed. Near expiry fentanyl was in use	Deferred the use of near expiry medications because of the risk of decreased or no efficacy
No effect of inhalational agent observed	2	Empty vaporizer of isoflurane discovered	Machine and medication check by anesthesia technicians per shift three times per day Low flow anesthesia stressed to prevent repeated emptying of vaporizers *“Quick machine checklist to be introduced before every case”
Bradycardia and apnea after inter-scalene block administrated	1	Intravascular injection of local anaesthetic	Training of the faculty and residents for Ultrasound guided blocks started in year 2007
Patient developed sudden apnea after spinal anesthesia initiated	1	Atracurium given I.V. instead of Mz. Patient was immediately sedated, trachea intubated, and ventilation initiated	Mz was removed as stock items and physician order entry was made mandatory to get Mz *“To revisit the standardization of syringes”
Atracurium administered instead of saline flush	3	Color coded labels were not available. Five ml syringe was used for both atracurium and saline flush	Availability of color coded labels was ensured
No response to treatment after severe hypotension	4	Phenylephrine, diluted and dispensed by pharmacy was not working. New medication prepared and administered	Pharmacy services for the dilution of phenylephrine and ephedrine was withdrawn
SucC was accidently used to flush the I.V. cannula	1	Deviation from routine practice. Mistake was immediately recognized Patient was immediately sedated and trachea intubated	Separate printed saline flush labels were made available after the incident Practice of reading out loud before giving any medication instituted
Patient found unconscious with oxygen saturation of 45% in recovery room. He had been irritable because of Foley’s catheter insertion	1	Mz was administered without POE. Patient regained consciousness after use of flumazenil	Mz was removed from stock items
Papaverine given through I.V. route by relieving staff	1	No formal handover given by primary anesthetist to the reliever. Antibiotic infusion was being administered. After finishing antibiotic, reliever injected Papaverine	Medication trolley is only meant for preparation of I.V. Medication for local use by the surgeon shouldn’t be place on anesthesia medication trolley. This was communicated to surgical teams
Oxytocin drip started before start of C-section	1	Deviation from practice	Point reiterated in the CI meeting that do not attach Oxytocin drip before it is indicated
Patient did not have muscle relaxation after SucC administered	1	Phenylephrine administered instead of SucC	Alert labels to be put on look-alike drugs
Severe bradycardia and hypotension requiring treatment with Glycopyrronium and atropine	1	Patient was already taking calcium channel blocker; induction was with sevoflurane and I.V. lignocaine	Awareness of drug interaction through CI meeting
Patient required re-intubation after extubation	1	After tracheal extubation cannula was flushed with muscle relaxant instead of saline	Readout loud before use of any medication *“Introduce syringe with color coded plungers”
Patient was not paralyzed after giving SucC but became tachycardiac (120/min)	1	Epinephrine filled in syringe instead of SucC. The alert label was on the top of the ampoule so once opened, it created issue of look-alike drugs	Emphasized that practice should be to break one ampoule, fill it, label it and then take the new one

*M* medication, *Mz* Midazolam, *SucC* SuccinylCholine, *POE* Physician Order Entry, *Future Plan.

## Discussion

Medication errors are defined as any preventable event that may cause or lead to inappropriate medication use or patient harm while the medication is in the control of the health care professional, patient or consumer. This chart review identified 311 drug related incidents (30.9% of total 1006 reported incidents) over a period of fifteen years. Human error contributed to 66% of the MEs and 32% of the errors showed a deviation from standard practice. Medication handling, and administration accounted for 60% of the reports. Thirty eight percent of the errors resulted in harm to the patients. Out of 86 ADEs reported there were 23 serious and 5 life-threatening errors. The actual harm that came to the patients was 1.6%. Neuromuscular blockers, narcotic analgesics, sedatives, and vasopressors contributed in 67% of the errors.

There are different methods to detect and report medication errors or ADEs, like self-reporting, incident reporting, manual chart review, automated computerized surveillance, or direct observation. Each has its value and limitations, but all highlight the problematic areas that need attention. Critical incident analysis is a process of collecting and reviewing reports in a way that helps in identifying trends in terms of frequency and harm. The process identifies contributing factors, and is helpful in education, research, development of policies, guidelines budget and planning, to provide safe anesthesia care^10,11^. It is a low-cost measure and of value in LMICs.

Our hospital serves as a tertiary care center in the area. A CI reporting program was initiated in our department in year 1995. These incidents are periodically reviewed and presented in departmental academic meetings where staff is also reminded on how to and when to fill the CI forms^12^. Once the error is reported the root cause must be analyzed, preventive measure instituted and shared with others. Quality Improvement Issues (QII) meetings were initiated in the department in year 2000, where errors were selected for taking further action and corrective strategies were prioritized. All incidents are reported anonymously on voluntary basis. This simple quality improvement and risk reduction measure helped us in improving the standard of anaesthetic care in our setup with low resources, where the lowering cost is an important aspect of implementing new safety measures^13^.

Several corrective strategies related to medications were put in place. Strategies that had significant effect were standardization of syringe sizes for specific drugs, and changes in printed self-adhesive labels where the drug name and concentration were made bold. Another additional strategy was the change in the floor stock which is present in operation room “drug trolley” that was reduced in quantity for some medications and temperature sensitive medications were kept in refrigerator with the provision of instant availability. The supervision of trainees for drug dilutions was made more stringent by trainers, and a file “standard dilution of vasoactive medication” was made available in the operating room (OR) suite. It was also observed that one person responsible for all phases of medication handling was an error reduction strategy and was recommended. Syringe standardization for different medication had already been in place in the department but CI still resulted due to deviation from the standards. One of the possible reasons of this deviation was the “subjective practices” without following the set standards. One of the steps in reducing such errors was regular reminders and presentation in departmental CI meetings which were followed by a “lesson to learn” e mail generated by the departmental CI coordinator after each meeting and shared with all staff.

Two third of the MEs were classified as human errors (HE). These usually happened because of personnel preferences and non-adherence to existing standard processes. The hospital also switched the manuals of policies and guidelines from hard copies to the online version during 2010 to 2015. All new inductees in the department were also instructed during the orientation program to go through these online resources. There is still no formal sign off that they have read and understand. It could be one of the reasons of deviation from practice when systems in place are not followed properly. An example of this is improvement in system like color coded labels does not ensure further decrease in labelling error or bring it to “ZERO” and this is the result of deviation from standard practice. The future task is to ensure compliance to this step. Repetition of HE after corrective measures have been put in place is regarded as negligence. Its recurrence can only be prevented by sharing these reports, educating, training, and updating the existing staff as well as new employees in the department.

As regards phase of drug handling, over-dosage was the most common administration error followed by wrong medicine, in-efficient medication, and under-dosage. The common causes of 38 cases of over-dosages were misunderstanding either verbal orders or deviation from standard dilutions. Other reason for overdose was the wrong dilutions in prefilled vasopressor syringes by pharmacy. After four MEs it was reported, immediate action taken, and the prefilled syringes were discontinued. Since then reverted back on our previous standard of dilution by ourselves. This is like what was reported by Sakagudin et al. who observed communication error as a main cause rather than lack of knowledge^14^. They rectified such errors by standardization of oral instructions (for e.g., “Inject 1.5 cc, 6 mg of Vecuronium out of ampoule containing 4 mg/cc”.) and making it a rule to repeat the given instructions^14^_._

The causes of administration of wrong medication in our study were syringe swap or ampoule swap and deviation from syringe standardization. There are two common mechanisms responsible for error during administration processes. The first was during preparation of the medication syringe from drug vial/ampoule by choosing wrong ampoule and diluting it to an unintended concentration. The second possibility was by accidentally picking up of wrong syringe/ampoule i.e., “syringe swap” which may happen because of distraction, inattention, or heavy workload. An observation was that breaking all ampoules at the same time and then filling all the syringes increased the risk of filling wrong medication in a syringe as well as label swap, it was reinforced to open and fill ampoules one by one. Lobaugh et al. reported an incidence of administration errors (65%) close to our findings but studied it in pediatric cases^15^. In contrast Sanduende et al. documented 42% errors during this phase^16^. Use of prefilled syringe has decreased preparation errors in some places^17^. We practice this for a few medications like opioids, but the rest are prepared by the anesthesiologist pre- induction. However, from the LMIC perspective it needs to be remembered that there is an additional cost involved in provision of pre-filled syringes.

Labelling also played important role in these errors. There is a controversy whether color coding of labels decreases or increases MEs. It was also observed that some incidents happened at times of shortage of color-coded labels resulting in use of white stickers which resulted in wrong medications being administered in 23 reports. Cheeseman et al. noted that addition of color to labels increased the speed of recognition; while Haslam et al. state that the process of implementing the International Color-Coding System increased their rate of medication errors due to a change in the system^18,19^_._ In our experience there was a decrease in ME after the introduction of standardized color-coded labels for frequently used medications in year 2007 as shown in Fig. 2. These colored labels were initially only applicable to medications that were prepared by anesthesiologists in OR. In 2018 our department adopted international color-coded labels for all medications used. Abeysekera et al. recommended further investigations to determine if color coded printed labels were effective in reducing medication error^20^.

According to a report by the Australian Incident Monitoring Study, neuro-muscular blockers (NMB) and opioids were the most frequently administered drugs in cases of wrong medication^20^. We also observed the same trend in our study, though NMBs were the highest in number (n = 102), but one of these were “ineffective medicine” where response of medicine was not achieved after a full calculated dose. Another impact of this inefficacy of NMBs is decreased satisfaction of surgical colleagues as well as frequent repeated doses and increase in cost. Action was taken by the pharmacy in ensuring cold chain and change of vendor (shown in Table 2). In contrast to our findings Kentaro et al. reported opioids and cardio-stimulants/vasopressors as the most common medications found in their study^14^.

The harm secondary to an ME can vary from minor physiological disturbance to life threatening morbidity and mortality. The incidence reported in literature varies from 0.01 to 11%^9,21,22^. In our patients this figure was 1.6% without any mortality. This was similar to a Brazilian study by Thomas et al. where they found morbidity and mortality with irreversible damage in 1.75% patients^2^. Nanji et.al from USA also reported a similar incident (1.6%) of life-threatening events, none were fatal^3^.

In order to prevent ME one needs to improve knowledge, increase reporting and sharing of incidents, vigilance; simulation-based teaching, orientation of the set standards to the new inductees in the department and development of clear communication. In 2019 Nanji identified several such strategies and further updated it in 2020 based on the recommendation of multi-regional associations for patient and medication safety to prevent perioperative MEs and/or ADEs. These were based on technology solution, standardization, elimination of lookalike medication vials and labels, pharmacy solution, and improvement in institutional culture^23^. Keeping the limitations of LMIC in mind we propose cost effective process-based interventions. Whether one uses technology or process, the first and foremost thing is to strengthen, design and comply with the processes of institution and the existing guidelines. Medication lay out is important to prevent syringe swaps and we applied it by keeping all cardiac medications in a separate bin at a separate place and it worked well. High alert labels on medication were one of the strategies that we found effective in preventing MEs.

Based on our review, we plan to introduce some further strategies within the department. One of these is an anesthesia drug checklist before every case, revision of syringe size for sedatives, implementation of change of practice to break, fill and label one ampoule at a time, before breaking the second ampoule, and introduction of syringes with color coded plungers. We also plan to introduce medication safety workshops based on common incidents, at least once a year. We are also deliberating whether to form a group or committee to monitor medication errors and provide weekly pictorial alerts. All new trainees, faculty members and technicians must go through all the guidelines and policies during orientation week and a simple quiz can be developed to certify that they have read as well as understood.

There are certain limitations to our study: firstly, it is a single Centre observational study, reporting was voluntary, and it was a retrospective review of a database. This could miss some unreported incidents as well as factual details of reports. CIR has its own limitation like under reporting, physician bias and their own perspective in the report^24^, lack of denominator, lack of sensitization of the value of reporting and delayed action after group discussion.

## Conclusion

Our review has revealed that medication errors are frequently occur during conduct of anesthesia with a high proportion due to human error. MEs mostly occurred at the time of administration. Clear and close loop communication and read the label twice, verbalize the medication going to be injected are few basic steps to control MEs during administration. Although many are readily caught and corrected, one tenth resulted in serious and life-threatening outcomes. Sharing of incidents during CI meetings and following it by a lesson to learn e-mail, introduction of color-coded labels and high alert labels, change in medication trolley lay out, were some of the low-cost strategies put in place to reduce incidents. This study thus shows the importance of medication error reporting as an initial step towards documenting MEs and using this information to devise preventive strategies.
